# Allergic adverse events following immunization: Data from post-marketing surveillance in Apulia region (South of Italy)

**DOI:** 10.3389/fimmu.2023.1074246

**Published:** 2023-02-27

**Authors:** Pasquale Stefanizzi, Davide Ferorelli, Francesco Livio Scazzi, Antonio Di Lorenzo, Andrea Martinelli, Chiara Trinchera, Lorenza Moscara, Enrico Miniello, Danilo Di Bona, Silvio Tafuri

**Affiliations:** ^1^ Bari Policlinico General Hospital, Interdisciplinary Department of Medicine, Bari, Italy; ^2^ Bari Policlinico General Hospital, Department of Emergency and Organ Transplantation, Bari, Italy

**Keywords:** vaccination, allergic events, anaphylaxis, AEFIs, surveillance, reporting rate

## Abstract

**Introduction:**

Among adverse events following immunization (AEFIs), allergic reactions elicit the most concern, as they are often unpredictable and can be life-threatening. Their estimates range from one in 1,000,000 to one in 50,000 vaccine doses. This report describes allergic events following immunization reported from 2020 to 2021 in Puglia, a region in the South-East of Italy with around 4 million inhabitants. Its main objective is to describe the allergic safety profile of currently employed vaccines.

**Materials and methods:**

This is a retrospective observational study. The study period spanned from January 2020 to December 2021, and the whole Apulian population was included in the study. Information regarding AEFIs reported in Puglia during the study period was gathered from the Italian Drug Authority’s pharmacovigilance database (National Pharmacovigilance Network, RNF). The overall number of vaccine doses administered was extrapolated by the Apulian online immunization database (GIAVA). Reporting rates were calculated as AEFIs reported during a certain time span/number of vaccine doses administered during the same period.

**Results:**

10,834,913 vaccine doses were administered during the study period and 95 reports of allergic AEFIs were submitted to the RNF (reporting rate 0.88/100,000 doses). 27.4% of the reported events (26/95) were classified as serious (reporting rate 0.24/100,000 doses). 68 out of 95 (71.6%) adverse events were at least partially resolved by the time of reporting and none of them resulted in the subject’s death.

**Conclusions:**

Allergic reactions following vaccination were rare events, thus confirming the favourable risks/benefits ratio for currently marketed vaccines.

## 1 Introduction

Vaccination is currently considered the most effective tool to prevent infectious diseases. During the 20th century, routine immunization determined a significant reduction in morbidity and mortality caused by infectious diseases such as poliomyelitis and smallpox, and all countries in the world adopted specific immunization strategies, in particular for newborns and infants (1).

As all drugs, though, vaccines can determine adverse events, which the American Food and Drug Administration defines as “any untoward medical occurrence associated with the use of a drug in humans, whether or not considered drug related” (2). Among these, allergic reactions are the ones that elicit the most important concerns in the general population, as they are often unpredictable and can be life-threatening (3–5). Their estimates range from one in 1,000,000 to one in 50,000 vaccine doses, and they are therefore classified as rare adverse events following immunization (AEFIs) (6–8).

Among allergic events, anaphylaxis is “a serious, generalized or systemic, allergic or hypersensitivity reaction that can be life-threatening or fatal” (9). It is a severe form of IgE-mediated reaction, which can be fatal and generally manifests minutes after the product’s administration, despite sometimes occurring hours after vaccination (10).

Most episodes of anaphylaxis are characterized by cutaneous symptoms of urticaria and angioedema, which may however be absent in 10-20% of the cases (11). Other symptoms include: respiratory manifestations of dyspnea, wheeze/bronchospasm, stridor, hypoxemia and/or decreased peak expiratory flow; cardiovascular phenomena including decreased blood pressure, possibly leading to end organ dysfunction; gastrointestinal symptoms including vomiting, crampy abdominal pain and diarrhea (12). It is relevant to consider that hypotension may not be detected in infants during anaphylaxis (13). Current estimates of anaphylaxis occurrence suggest that it is an extremely rare phenomenon, ranging from one in 100,000 to one in 1,000,000 doses (4).

In Italy, reporting AEFIs is mandatory for all healthcare workers observing them, as per Regulation N° 1235/2010 of the European Parliament (14). The patients themselves may report adverse events they have suffered *via* the dedicated section of the Italian Drug Authority (AIFA) website. When signaling an AEFI, the reporting subject is requested to classify it as serious or non-serious, keeping into consideration that serious AEFIs are defined as events that result in the subject’s death or life danger, significant and/or permanent impairment, hospitalization or prolongation of current hospitalization, congenital anomaly or birth defect, or that require intervention to prevent said damages (15). Additional medical conditions that are classified as serious AEFIs are the ones reported in the list of important medical event terms developed by the European Medicines Agency’s EudraVigilance Expert Working Group (16).

Following each report’s reception, regional pharmacovigilance supervisors are required to carry out causality assessment on all serious adverse events, following the recommendations provided by the World Health Organization (WHO). This standardized approach aims to increase the quality of surveillance, as well as to avoid emotional biases which may influence the public’s perception of vaccine safety and increase vaccine hesitancy (17, 18).

This report describes allergic events following immunization reported from 2020 to 2021 in Puglia, a region in the South-East of Italy with around 4 million inhabitants. Its main objective is to design the allergic safety profile of currently employed vaccines.

## 2 Materials and methods

This is a retrospective observational study. Data regarding the overall number of vaccine doses administered from January 1st, 2020, to December 31st, 2021, were gathered from the regional online immunization database (GIAVA). The National Pharmacovigilance Network (RNF), AIFA’s database for adverse event surveillance, was the data source for allergic AEFIs reported during the study period. RNF uses the Medical Dictionary for Regulatory Activities’ (MedDRA) coding system for AEFIs.

Cases of allergic events following immunization were identified *via* the following algorithm:

“case” IF wheals OR erythema OR allergy OR angioedema OR rash OR urticaria OR itch OR allergic reaction OR anaphylactic shock NOT injection site wheals.

This algorithm was obtained by confronting all its items with those included within the item list from MedDRA. By using the algorithm, all items responding to the definition of “allergic reaction” in MedDRA were successfully identified and therefore included into the research. Injection site wheals were excluded due to the high probability of them being related to the vaccine’s administration rather than to the vaccine itself.

For every subject who suffered from allergic reactions following vaccination, information was collected about date of birth, sex, date of vaccine administration and other vaccines administered on the same date. AEFIs’ descriptions included the following data: date of onset, date of computing in RNF, clinical characteristics, duration, treatment, outcome, hospitalization or emergency room access, and a description of the case.

Classification of AEFIs as “serious” or “non-serious” was performed following WHO guidelines. Serious AEFIs were furtherly classified following causality assessment as having a “consistent causal association” or “inconsistent causal association” with the vaccination, as “indeterminate” or “non-classifiable” (19). For AEFIs that required hospitalization, medical records were taken into consideration as well in order to obtain additional information. Causality assessment was carried out by two different physicians with expertise in vaccinology by confronting serious adverse events with the relevant literature concerning allergic AEFIs. Results were therefore compared. In case of divergent opinions literature was reviewed, and a third physician was consulted.

The database was built using software Microsoft Excel^®^. Reporting rates were calculated as the number of AEFI reports during a certain time span divided by the vaccine doses administered during the same period. Statistical analysis was performed *via* software Stata MP17^®^. Categorical variables were expressed as percentages and compared *via* Chi-squared test. A p-value of 0.05 was chosen as a break point for statistical significance.

## 3 Results

10,834,913 vaccine doses were administered during the study period and 5,145 reports of AEFIs were submitted to the RNF (reporting rate 47.5/100,000 doses), 95 of which concerning allergic AEFIs (reporting rate 0.88/100,000 doses). 81.1% of the reported AEFIs (77/95) occurred in women, and the mean age of subjects who reported one or more AEFIs was 40 ± 20.9 years (minimum 3 months, maximum 88 years).

The most common adverse events reported with allergic reactions after vaccination were urticaria/angioedema (68 events), itching (19), wheals (18), hyperpyrexia (13) and pain or tenderness near the injection site (11). A full list of reported adverse events is provided in **Table 1**, while the outcome of all 95 adverse events is reported in **Table 2**. For 69.5% (66/95) of reported AEFIs, at least partial healing occurred by the time data was collected. 21.0% of them (20/95) had yet to recover, while an additional 2.1% (2/95) resulted in residual signs or symptoms despite the subject having recovered. For seven reports (7.4%) no outcome was notified.

**Table 1:** Reported adverse events after immunization, per sign/symptom type (note that percentages do not add up to 100%, since one reporting may count more than one adverse event).

Type of AEFI	N°	%	Reporting rate/100,000 doses
Urticaria/angioedema	68	71.6	0.63
Itching	19	20.0	0.18
Wheals	18	18.9	0.17
Hyperpyrexia	13	13.7	0.12
Local pain/tenderness	11	11.6	0.10
Dyspnea	9	9.5	0.08
Skin rash	9	9.5	0.08
Erythema	8	7.4	0.07
Headache	7	7.4	0.06
Urticaria-like skin rash	6	6.3	0.06
Asthenia	5	5.3	0.05
Myalgia	5	5.3	0.05
Oedema	5	5.3	0.05
Anaphylactic shock	2	2.1	0.02

**Table 2:** Outcome of all reported AEFIs.

Outcome of AEFI	N°	%
Fully healed	46	48.4
Improvement	20	21.0
Not yet healed by the time of reporting	20	21.0
Non-available	7	7.4
Healed with residual signs/symptoms	2	2.1

27.4% of the reported events (26/95) were classified as serious (reporting rate 0.24/100,000 doses). The proportion of serious adverse events detected among women was 23.4% (18/77), while it was 44.4% in men (8/18; p=0.0726). Following causality assessment, 96.1% (25/26) of said serious events were deemed consistently causally associated with the vaccines’ administration, while the remaining AEFI was classified as “indeterminate” due to insufficient information provided by the reporting subject. The reporting rate for vaccine-related serious adverse events was therefore 0.23/100,000 doses.

Two cases of anaphylactic shock were reported (reporting rate 0.02/100,000 doses), one following Comirnaty^®^ administration and the other following Pneumovax^®^ administration. The former occurred in a 45-year-old female patient with a history of allergic reactions who was vaccinated in a sheltered environment but with no premedication; minutes after immunization, the subject started lamenting loss of sensitivity to the face and the lips, speech and movement impairment. Confusion, balance loss, tachycardia and blood pressure increase followed, requiring intervention by the medical personnel; intravenous corticosteroid and intramuscular antihistaminic were administered, causing the patient to recover. However, the subject accessed the local emergency room multiple times during the following days due to persistent bronchospasm and required multiple intramuscular corticosteroid injections in order to fully recover from the episode. On the contrary, the Pneumovax^®^-related anaphylactic event occurred in a 78-year-old male who experienced intense dyspnea and thorax constriction feeling, followed by the rapid onset of erythema and profuse sweating. The subject was treated immediately with adrenaline and corticosteroid administration but did not require either hospitalization or emergency room access, as clinical conditions improved rapidly after treatment.

The outcomes of the 26 serious adverse events are reported in **Table 3**. It should be noted that none of the reported adverse events resulted in death or permanent and significant impairment.

**Table 3:** Outcome of serious reported AEFIs.

Outcome of serious AEFI	N°	%
Fully healed	13	50.0
Not yet healed by the time of reporting	6	23.1
Improvement	3	11.5
Non available	3	11.5
Healed with residual signs/symptoms	1	3.8

**Tables 4**, **5** synthetize the number of allergic AEFIs and serious allergic AEFIs reported for each vaccine during the study period, respectively.

**Table 4:** Number and Reporting Rate of allergic AEFIs, per vaccine.

Vaccine	N°	%	Reporting rate/100,000 doses
Comirnaty	51	53.7	0.95
Vaxzevria	14	14.7	1.60
Spikevax	10	10.5	0.76
Bexsero	8	8.4	3.93
Vaxelis	2	2.1	3.26
Flucelvax Tetra	2	2.1	2.44
Fluad	2	2.1	0.26
Pneumovax	1	1.1	4.71
Proquad	1	1.1	0.93
Janssen	1	1.1	0.92
Gardasil 9	1	1.1	0.80
Vaxigrip Tetra	1	1.1	0.09

**Table 5:** Number and Reporting Rate of serious allergic AEFIs, per vaccine.

Vaccine	N°	%	Reporting rate/100,000 doses
Comirnaty	16	64.0	0.30
Vaxzevria	5	20.0	0.57
Pneumovax	1	4.0	4.71
Vaxelis	1	4.0	1.63
Bexsero	1	4.0	0.49
Fluad	1	4.0	0.13

## 4 Discussion

During the study period 95 reports of AEFIs involving one or more symptoms of allergic reaction were submitted in Puglia *via* passive surveillance activities. The reporting rate for allergic events following immunization was therefore 0.88/100,000 administered vaccine doses. Urticaria was the most common manifestation, and 26 out of 95 AEFIs were classified as serious (reporting rate 0.24/100,000).

According to our data, allergic reactions following vaccination are a rare occurrence, since less than one person out of 100,000 receiving a vaccine dose is expected to suffer from any of these phenomena. Serious AEFIs are even less common, representing little more than a quarter of all reported allergic reactions, and none of them led to either the patient’s death or serious and permanent impairment (Table 3). On the other hand, most of the reported serious adverse events were deemed related to the vaccines’ administration. It should also be noted that no vaccines showed especially high allergic AEFI reporting rates (Table 4).

A 2015 study by McNeil et al. estimated the incidence of anaphylaxis after vaccination at 1.31 (95% confidence interval, 0.90-1.84) per million doses (20). Such results are fairly similar to our findings regarding anaphylactic events, despite McNeil’s study population being much larger than ours (over 25 million vaccine doses administered over a two-year time span). This might be caused by the nature of allergic adverse events, which are sudden, have an apparent manifestation and are often perceived as an immediate threat to the patient’s survival, even when this is not the case (11–13, 21). Such events may therefore be more likely to be reported either by healthcare professionals or by the patients themselves.

A 2019 narrative review by Stone et al. confirmed the infrequent nature of immune-mediated adverse reactions to vaccines. Despite including non-allergic manifestations such as Guillain-Barré syndrome in their paper, Stone and colleagues observed that the cumulative incidence of these phenomena is lower than one case in a million vaccine administrations (22).

A far smaller, yet interestingly insightful study was conducted by Micheletti et al. in 2012. The group reviewed cases of allergic reactions or contraindications to vaccines submitted to the “Green Channel”. The Green Channel is a University Hospital Immunization Consultancy Clinic operating in the Veneto region (Northern Italy) and specialized in immunization counseling for both healthcare workers and individuals with a history of allergic reactions or other contraindications. During the study, 1,425 subjects submitted to the clinic and 519 (36.4%) were sorted out for suspected allergy to vaccines. Out of these patients, more than 85% were subsequently deemed eligible for vaccination following evaluation of their clinical records and/or sensitivity testing (23).

Allergic events observed in the Green Channel Consultancy Clinic were much more frequent than the ones reported in McNeil’s and Stone’s studies, even when eliminating those mistakenly identified as allergic reactions. Our study, too, highlighted a greater proportion of allergic AEFIs. This observation is explained by the nature of Micheletti’s sample population, which consisted of subjects who had already reported one or more episodes of vaccine allergy. It is however interesting to notice how much the epidemiology of allergic reactions is influenced by the context in which data is collected.

The main strength of our study is represented by the numerosity of the population we addressed. Our data refers in fact to the whole Apulian population, which is currently over 4 million inhabitants. Moreover, even though the study period was relatively short, no specific vaccine was investigated and therefore over 10 million administrations were considered. Such size is significantly larger than that usually represented in clinical trials.

In addition to this, by considering the causality assessment’s outcomes for serious AEFIs we addressed the issue of causal association between vaccines and adverse events following their administration. This kind of approach is currently recommended by WHO, and allows to clarify whether the AEFI signal is clear or distorted by background noise. It is therefore considered paramount for post-marketing studies (24).

Nevertheless, our study is partially weakened by the passive surveillance method employed by AIFA’s pharmacovigilance network. Under-reporting is in fact a well-known phenomenon in passive surveillance systems, as healthcare personnel tend to overlook mild and self-limiting adverse events while reporting serious AEFIs more accurately (25–27). Despite this flaw, large surveillance networks have to rely on passive surveillance, as active data collection is not feasible when confronting vast populations. Finally, it should be mentioned that our study is a retrospective one, and is therefore intrinsically limited in its design.

In recent medical history, vaccines’ safety profiles have become an increasingly important subject. Vaccine hesitancy is often a complex and multi-factorial phenomenon which burrows its roots into the subjects’ fear of what harm may come from substances they do not fully understand (28, 29). A history of allergic reactions is likely to engender a certain degree of apprehension, as the patient to be vaccinated may expect to suffer from adverse events. Vaccine-related anxiety may cause adverse events to ensue, thus compromising the subject’s compliance to future immunization programs as well as producing the aforementioned noise in the AEFI signal (29, 30).

These concerns are especially relevant in the contemporary post-pandemic scenario. In fact, the Severe Acute Respiratory Coronavirus 2 (SARS-CoV-2) pandemic has been limiting the activities of vaccination services all over the world for the last three years, leading to a significant decrease in vaccination coverage, especially in children (31). According to recent data from the United Kingdom, for example, delays were observed as far as the anti-Measles-Mumps-Rubella (MMR) paediatric vaccination is concerned. This has caused MMR coverage to plummet below 90%, leading to an increase in the risk of Measle epidemics (32).

Allergic reactions are hardly ever reported in clinical trials, as study samples are usually recruited keeping into consideration the subjects’ medical records. Therefore, people with a history of allergies and/or anaphylaxis are often discarded before the trial’s beginning. It is therefore of utmost importance to investigate into such matter with special attention during the post-marketing life of vaccines, while also communicating with clear and easy-to-understand words the real risks and benefits deriving from vaccination.

This subject is especially important in the context of the currently ongoing anti-SARS-CoV-2 vaccination campaigns: in fact, since these vaccines have been marketed, there have been numerous reports of severe allergic reactions. The risk of potentially serious allergic adverse events therefore represents a strong concern among the public and must be addressed properly (33, 34). Our data shows that allergic reactions are a niche phenomenon, and therefore do not compromise the safety profile of the currently marketed vaccines: communicative and educational strategies directed to target subjects and healthcare workers have to be implemented to improve confidence and vaccine uptake.

## Data availability statement

The data analyzed in this study is subject to the following licenses/restrictions: The data employed in our research is part of the National Pharmacovigilance Network, and as such is available exclusively by request of credentials to the Italian National Drug Authority (AIFA). Requests to access these datasets should be directed to pasquale.stefanizzi@uniba.it.

## Author contributions

PS and DF designed the study and drafted the manuscript. LM and EM contributed to the study design. FS and CT were responsible for data collection. AD and AM were responsible for data analysis. DD and ST revised the study protocol as well as the manuscript itself. All authors contributed to the article and approved the submitted version.
